# Carbon tetrachloride (CCl_4_) accelerated development of non-alcoholic fatty liver disease (NAFLD)/steatohepatitis (NASH) in MS-NASH mice fed western diet supplemented with fructose (WDF)

**DOI:** 10.1186/s12876-020-01467-w

**Published:** 2020-10-15

**Authors:** Guodong Zhang, Xiaoli Wang, Tzu-Yang Chung, Weiwei Ye, Lauren Hodge, Likun Zhang, Keefe Chng, Yong-Fu Xiao, Yixin Jim Wang

**Affiliations:** 1 Crown Bioscience (CBLA), New Iberia, Louisiana USA; 2 Crown Bioscience (CBTC), Taicang, China

**Keywords:** NAFLD, NASH, FATZO, High fat diet, Liver fibrosis, Hepatosteatosis, Inflammation

## Abstract

**Background:**

Multiple murine models of nonalcoholic fatty liver disease/steatohepatitis (NAFLD/NASH) have been established by using obesogenic diets and/or chemical induction. MS-NASH mouse (formally FATZO) is a spontaneously developed dysmetabolic strain that can progress from hepatosteatosis to moderate fibrosis when fed a western diet supplemented with 5% fructose (WDF). This study aimed to use carbon tetrachloride (CCl_4_) to accelerate and aggravate progression of NAFLD/NASH in MS-NASH mouse.

**Methods:**

Male MS-NASH mice at 8 weeks of age were fed WDF for the entire study. Starting at 16 weeks of age, CCl_4_ was intraperitoneally administered twice weekly at a dose of 0.2 mL/kg for 3 weeks or 0.08 mL/kg for 8 weeks. Obeticholic acid (OCA, 30 mg/kg, QD) was administered in both MS-NASH and C57Bl/6 mice fed WDF and treated with CCl_4_ (0.08 mL/kg).

**Results:**

WDF enhanced obesity and hepatosteatosis, as well as induced moderate fibrosis in MS-NASH mice similar to previous reports. Administration of CCl_4_ accelerated liver fibrosis with increased bridging and liver hydroxyproline contents, but had no significant impact on liver steatosis and lipid contents. High dose CCl_4_ caused high mortality and dramatic elevation of ALT and ASL, while low dose CCl_4_ resulted in a moderate elevation of ALT and AST with low mortality. Compared to C57BI/6 mice with WDF and CCl_4_ (0.08 mL/kg), MS-NASH mice had more prominent hepatosteatosis and fibrosis. OCA treatment significantly lowered liver triglycerides, steatosis and fibrosis in both MS-NASH and C57Bl/6 mice fed WDF with CCl_4_ treatment.

**Conclusions:**

CCl_4_ reduced induction time and exacerbated liver fibrosis in MS-NASH mice on WDF, proving a superior NASH model with more prominent liver pathology, which has been used favorably in pharmaceutical industry for testing novel NASH therapeutics.

## Background

Nonalcoholic fatty liver disease (NAFLD) is a prevalent complication of metabolic diseases, comprising a cluster of conditions spanning from early hepatic steatosis to late stage cirrhosis in the absence of alcohol consumption [1, 2]. While simple steatosis with minimal inflammation has no clinical implications, nonalcoholic steatohepatitis (NASH) with lobular inflammation has serious consequences as it progresses to liver fibrosis in 10–20% of cases, leading to cirrhosis and possible hepatocellular carcinoma (HCC) [3]. A sequential two- or multiple hit model of pathogenesis was proposed for the progression of liver steatosis to NASH; first, hepatic fat accumulation results in both macrovesicular (adipocyte accumulation) and microvesicular (hepatocyte ballooning) steatosis [4, 5], followed by exposure of the accumulated hepatic lipids to hepatic oxidative stress (lipid peroxidation to release lipid peroxides) [6, 7], which causes inflammatory infiltration, finally, hepatocyte damage, repairment and fibrosis [8–10]. In addition, insulin resistance, adipose tissue-derived factors, nutritional factors, gut microbiota, and genetic and epigenetic factors, work together and contribute to the pathogenesis of NAFLD/NASH [5].

Multiple murine models of NAFLD/NASH have been developed using obesogenic or nutrient-deficient diets, chemical induction, genetic modification, or a combination of these manipulations [11–17]. MS-NASH mouse, formerly published as FATZO [18], was developed by crossing C57Bl/6 and AKR/J strains with selection for dysmetabolic phenotypes. The is a new generation of animal model that spontaneously develops obesity, metabolic disorders and hyperglycemia under a standard chow diet in the presence of an intact leptin signaling pathway. They begin to exhibit glucose intolerance, insulin resistance and hyperinsulinemia as early as 6 weeks of age, but does not spontaneously develop NASH/NAFLD [19, 20]. Western (high fat) diet supplemented with fructose (WDF) has been reported to induce NASH/NAFLD in murine models [11, 16, 21]. When fed WDF, MS-NASH mice develop NAFLD/NASH phenotypes with elevation of plasma ALT/AST and lipid as early as 4 weeks, an increase in liver triglycerides ~ 12 weeks, and exhibition of hepatic steatosis, ballooning, inflammation and mild to moderate fibrosis ~ 20 weeks [22]. In recent years, MS-NASH mice on WDF have been used as an improved translational model of obesity, metabolic disorders, diabetes, and NAFLD/NASH for drug discovery and development. However, the NASH induction time is relatively long (~ 20 weeks) with relatively moderate liver fibrosis (pathology score ~ 1).

Carbon tetrachloride (CCl_4_) is a well characterized liver toxin that causes direct hepatocyte injury, leading to liver fibrosis and HCC [23–25]. Administration of CCl_4_ in C57Bl/6 mice has been shown to cause liver fibrosis [26]. Furthermore, administration of CCl_4_, in high fat diet-induced obesity (DIO) model in C57Bl/6 mice resulted in histopathological features of NASH with increased serum ALT and liver hydroxyproline [27]. While useful for modeling some aspects of NASH, the C57Bl/6 strain lacks the features of dysmetabolism and diabetes, which typically accompany NAFLD/NASH etiology in humans [14, 15].

The aims of the present study are to use CCl_4_ in MS-NASH mice fed WDF to accelerate disease progression by reducing induction time and exacerbating liver fibrosis in a strain predisposed toward metabolic disorders, hepatosteatosis and other comorbidities of NASH/NAFLD seen in humans, such as obesity, insulin resistance, and diabetes.

## Methods

### Animals

Male MS-NASH mice (formally FATZO) [18] were developed by Crown Bioscience as a new generation of mouse model presenting high translatable phenotypes in human diseases such as obesity, metabolic disorder, diabetes and NAFLD/NASH [19, 20, 22]. The animals for this study were bred and then housed individually in IVC cages (Taicang, China) or open ventilated cages (Indianapolis, IN) with room temperature maintained at 22–26 °C, a 12-h light cycle (06:00–18:00), and distilled water ad libitum. The animals were fed control diet (CD, Purina 5008 chow, LabDiet, St. Louis, MO) for 8 weeks after birth, then stratified into different experimental groups based on body weight, serum ALT and AST. C57Bl/6 J mice (The Jackson Laboratory, Ellsworth, Maine) were used as control strain. All mice were maintained and treated in accordance with the guidelines of Association for Assessment and Accreditation of Laboratory Animal Care (AAALAC). Experimental protocols were approved by the Institutional Animal Care and Use Committee (IACUC).

### Effects of CCl_4_ in MS-NASH mice fed Western diet supplemented with fructose (WDF)

The first study aimed to, 1) confirm the characterization of MS-NASH mice fed WDF (40% kCal fat, 43% kCal carbohydrate, 17% kCal protein, D12079B, Research Diets, New Brunswick, NJ) to induce liver phenotypes; and 2) examine the dose effect of CCl_4_ to shorten the induction time and to enhance liver fibrosis.

The original CCl_4_ solution was further diluted in olive oil (Sigma Aldrich) at final concentrations of 0.02 and 0.005 mL/mL, with a dosing volume of 10 and 1.5 mL/kg, injected intraperitoneally (IP) twice weekly (BIW) at a final dose of 0.2 and 0.08 mL/kg for high and low dose group, respectively.

#### High dose CCl_4_ (0.2 mL/kg, BIW) for 3 weeks

After 8 weeks on CD, MS-NASH mice were divided into following 3 groups: 1) CD (*n* = 8): continued on CD for 11 weeks; 2) WDF (n = 8); and 3) WDF + CCl_4_ (*n* = 6): switched to WDF for the rest of 11 weeks to induce liver phenotypes; after 8 weeks on WDF, vehicle or CCl_4_ was injected IP, BIW for 3 weeks.

#### Low dose CCl_4_ (0.08 mL/kg, BIW) for 8 weeks

After 8 weeks on CD, MS-NASH mice were switched to WDF for 16 weeks to induce liver phenotypes. At 8 weeks after WDF, the animals were divided into 2 groups: 1) WDF (*n* = 4); and 2) WDF + CCl_4_ (*n* = 11), with IP injection of vehicle or low dose CCl_4_, BIW, respectively for the final 8 weeks.

### Effects of Obeticholic acid (OCA) in mice fed WDF plus CCl_4_

After 8 weeks on CD, MS-NASH or C57BI/6 mice were fed WDF for 16 weeks to induce liver phenotypes. After 8 weeks on WDF, the animals were injected IP with low dose CCl_4_ (0.08 mL/kg, BIW) and divided into vehicle (*n* = 11) and OCA (*n* = 10) groups for an additional 8 weeks during which, vehicle (1% methylcellulose, Sigma Aldrich) or OCA (Toronto Research Chemicals, New York, ON, Canada, 30 mg/kg) was administrated orally once daily. C57Bl/6 mice were compared with the same protocol in vehicle (*n* = 9) or OCA (n = 9) groups.

### Sample collection, processing and measurements

In all studies, body weights were recorded every 4 weeks. At the end of studies, all mice were euthanized by CO_2_ inhalation and with cervical dislocation approximately 24 h after the last CCl_4_ administration.

#### Blood samples

Blood samples were collected ~ 72 h after CCl_4_ dosing during the course of the experiment from the tail or ~ 24 h after the last dose of CCl_4_ at the end of the experiment via cardiac exsanguination, from which, serum was prepared for measuring AST and ALT by a clinical analyzer (Beckman-Coulter AU480; Brea, CA). A separate experiment was performed to measure the acute time course of ALT and AST at 24, 48 and 72 h in response to a single dose of CCl_4_ at 0.2 mL/kg in MS-NASH mice on CD.

#### Liver contents

The right lobe of the liver (~ 200 mg/animal) was collected and snap frozen in liquid nitrogen, placed in Lysing Matrix D Tubes with distilled water at 20% concentration (MP Biomedicals, Santa Anna, CA), and homogenized in a Fastprep-FP120 cell disrupter (Thermo Fisher Savant) in cold condition for 30 s. The liver contents of triglyceride and cholesterol were analyzed by a clinical analyzer (Beckman-Coulter AU480) within 30 min of sample preparation. Hydroxyproline was measured on the BioTek Synergy 2 Multi-Mode Microplate Reader utilizing a colorimetric assay kit (BioVision, Catalog #: K555–100) after hydrolyzed for 3 h at 120 °C at 1:1 ratio of sample homogenate to 12 N Hydrochloric acid (RICCA Chemical Co., Arlington, TX).

#### Liver histology

The left lobe of the liver was fixed in 10% neutral buffered formalin for 24 h followed by bath in alcohol then xylene for paraffin embedding, cut into 5-μm sections and stained with Hematoxylin and Eosin (H&E) and Picro Sirius Red (PSR). A whole slide digital imaging system (Aperio Scan Scope CS system, 360 Park Center Drive, Vista, CA) was used to scan the slides at 20x in 1.5 to 2.25 min.

### Liver histopathology analysis

#### Semi-quantitative scoring by a pathologist

Digital images were evaluated by a research pathologist blinded to different study groups with the standard NASH criteria for semi-quantitative scoring for hepatosteatosis, lobular inflammation, and hepatocyte balloon degeneration, respectively from H&E staining and then summated as a standard NAFLD Activity Score (NAS), commonly used in preclinical animal models and in patients [28, 29]. Fibrosis score was assessed systemically with pattern recognition from PSR staining. Three representative areas per liver were examined and the scores of each parameter from individual animal were averaged.

#### Computerized quantitative analysis

Computer software with automatic intelligence (AI) machine learning algorithm for histology analysis from Halo (Indica Labs, Albuquerque, NM) or ImageDx (Reveal Biosciences, San Diego, CA) were used to analyze digitally scanned images of H&E and PSR staining for quantitative analysis of steatosis, ballooning, inflammation or fibrosis in the same set slides evaluated by the pathologist. The analysis process included automated tissue identification, followed by segmentation of regions of interest for quantification of the following metrics: 1) Steatosis percentage: the area of total lipid accumulation subcategorized micro- or macro-vesicular within the entire section area; 2) Ballooning hepatocyte density: the density of ballooning hepatocytes within the entire section area; 3) Inflammatory cell density: the total number of inflammatory cells within the entire section area. All 3 parameters above were analyzed in the H&E stained section. 4) Fibrosis percentage: the total fibrosis area within the entire section area in the PSR stained section.

### Statistical analysis

All values are reported as mean ± standard error of mean (SEM), unless noted otherwise. Data were compared in MS-NASH mice on CD or WDF with or without CCl_4_; and effects of OCA were compared to vehicle with one-Way ANOVA for multiple groups or Holm-Sidak t-test for 2 groups. Survival curves of MS-NASH and C57Bl/6 mice treated with CCl_4_ were compared using Log-rank test for trend. Parametric correlation tests were conducted between pathologist scores and ImageDx quantitative analysis using Pearson correlation coefficient r. Statistical differences were denoted as two-sided *p* < 0.05 or *p* < 0.005. Prism software (GraphPad, version 8.3) was used for the statistical analysis and graphing.

## Results

### Dose effects of CCl_4_ in MS-NASH mice fed western diet supplemented with fructose (WDF)

#### High dose CCl_4_ (0.2 mL/kg, BIW) for 3 weeks

**Fig. 1:**
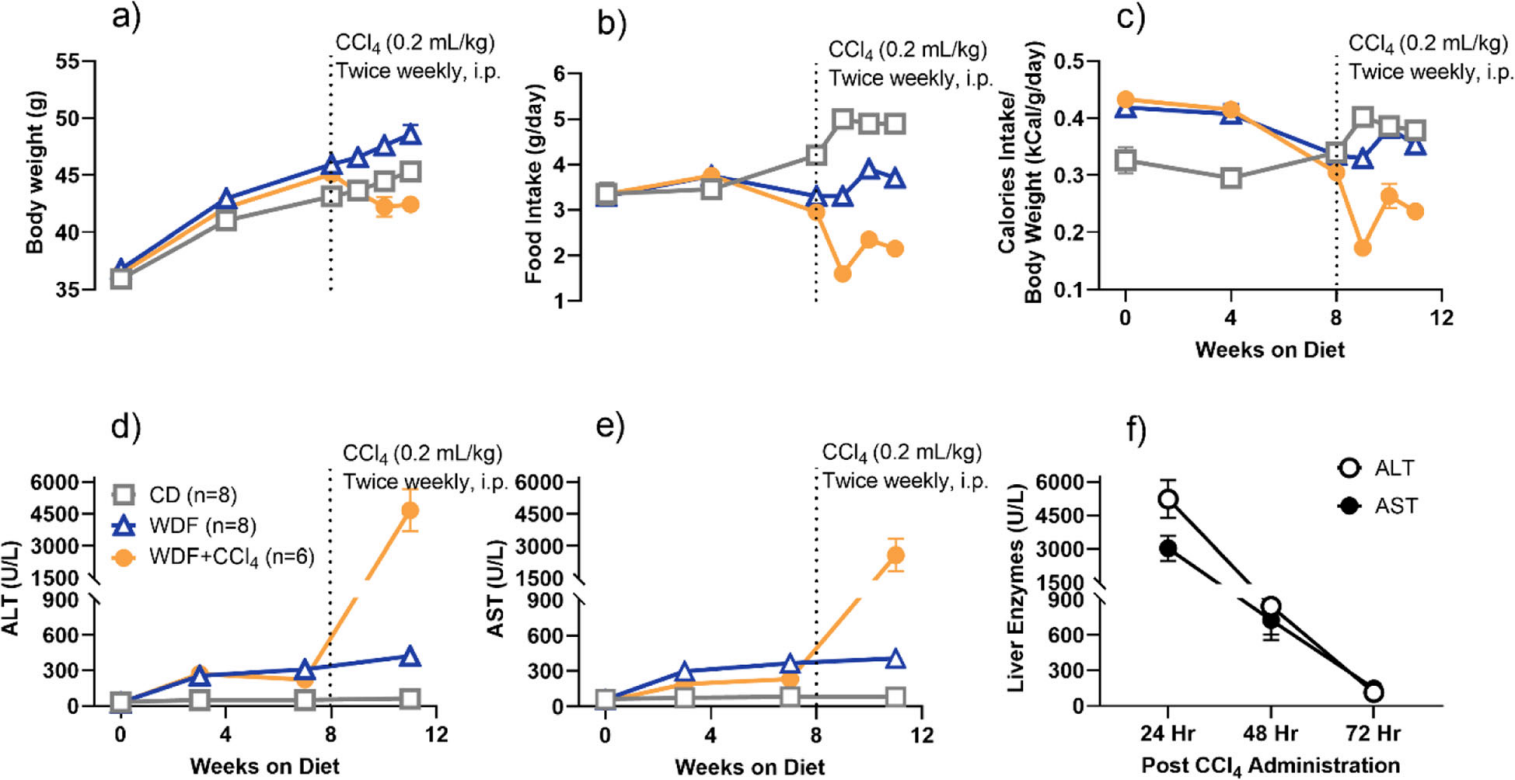
Effects of high dose CCL_4_ (0.2 mL/kg) twice weekly for 3 weeks in MS-NASH mice fed Control diet (CD) or Western diet supplemented with fructose (WDF). (**a**) Body weight; (**b)** daily food and (**c**) caloric intake; serum (**d**) ALT and (**e**) AST before and after repeated high dose CCl_4_. (**f**) Time course of acute response of ALT and AST to a single high dose CCl_4_ in mice on control diet (CD). Data presented as mean ± SEM

Similar to the previous report in MS-NASH mice [22], the present data confirmed that compared to the control diet (CD), WDF enhanced the obesity phenotype (Fig. 1a) with reduction in food (Fig. 1b), but not caloric (Fig. 1c) intake, and significantly elevated serum ALT (Fig. 1d) and AST (Fig. 1e).

To establish the proper dose of CCl_4_ that can accelerate disease progression and enhance liver fibrosis without significant toxic impact on MS-NASH mice, a dose of CCl_4_ at 0.2 mL/kg BIW was selected, which was a relatively low dose compared to those reported in many studies to induce liver fibrosis in normal rodents without steatosis [14, 23]. Compared to MS-NASH mice on CD or WDF without CCl_4_, administration of CCl_4_ significantly reduced body weight (Fig. 1a), food (Fig. 1b) and caloric (Fig. 1c) intake, as well as dramatically elevated ALT (Fig. 1d) and AST (Fig. 1e) measured ~ 24 h after CCl_4_ dosing.

In a separate group of MS-NASH mice fed CD, the acute response to a single dose of CCl_4_ at 0.2 mL/kg showed a similar elevation of AST and ALT at 24 h, but quickly diminished over the next 48 h (Fig. 1f).

**Fig. 2:**
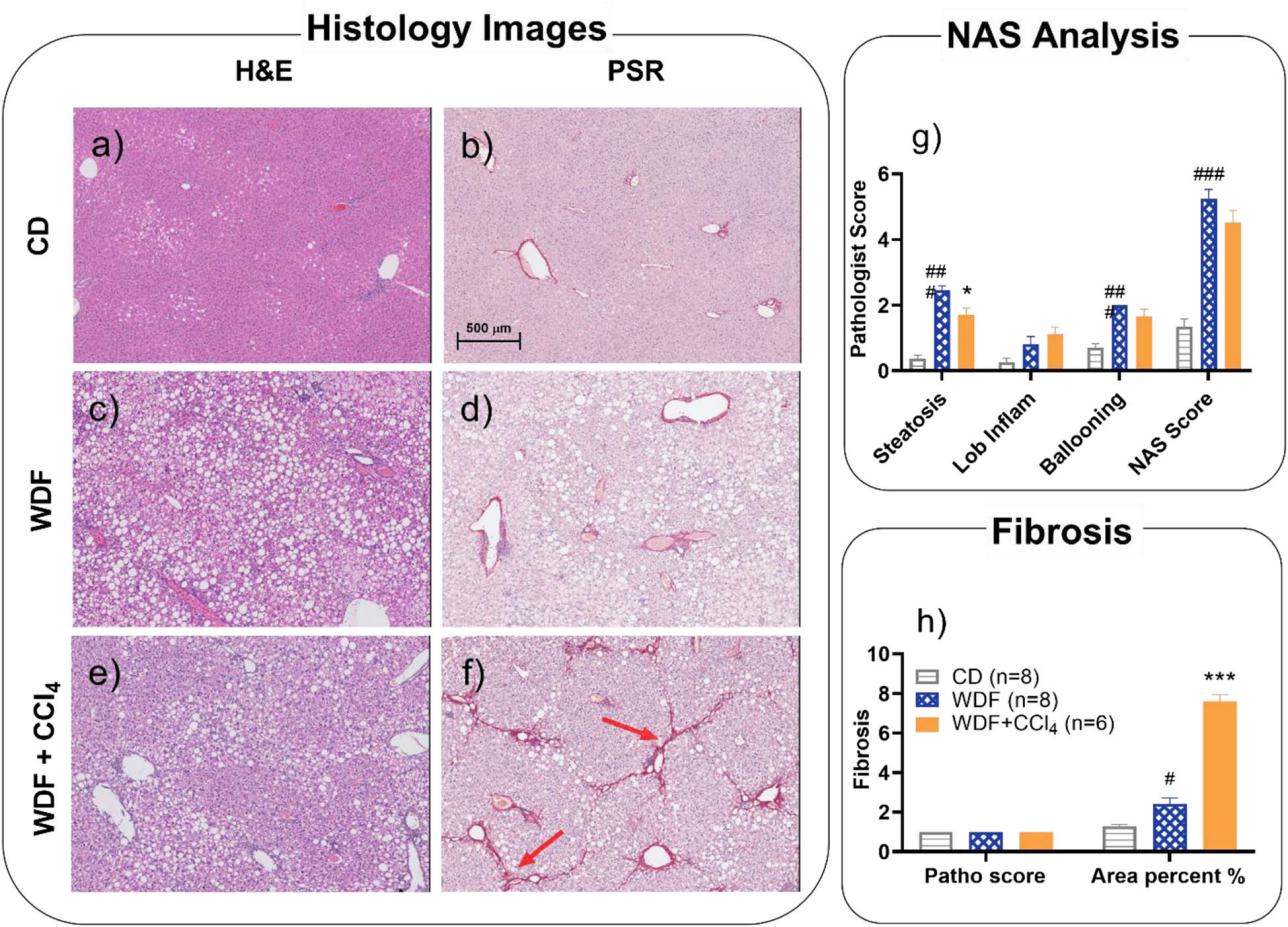
Histopathology in MS-NASH mice fed Control diet (CD) or Western diet supplemented with Fructose (WDF) treated high dose CCl_4_ (0.2 mL/kg) twice weekly for 3 weeks. Left panel: Representative images of H&E and PSR staining in animals fed (**a** and **b**) CD; and WDF treated (**c, d**) without or (**e, f**) with CCl_4_, respectively. Red arrows indicate fibrosis. Right panel: (**g**) Pathology scores of steatoses (0–3), lobular inflammation (0–3), ballooning (0–2), NAFLD Activity (0–8). (**h**) left: fibrosis score (0–4) and right: Percentage fibrosis area, quantitatively analyzed as total PSR positive staining area over total liver section area scanned and processed by HALO software. Data presented as mean ± SEM. # *p* < 0.05, ### *p* < 0.005 comparing with CD group; * p < 0.05, *** p < 0.005 comparing with WDF group by one-way ANOVA analysis

The representative histopathology images showed relatively normal liver tissue in MS-NASH mice on CD (Fig. 2a & b), but typical NAFLD/NASH pathology in MS-NASH mice on WDF with significantly increased macrovesicular fatty accumulation and microvesicular hepatocyte ballooning (Fig. 2c & d), which is similar to what we reported earlier [22]. Although Fig. 2e & f showed that CCl_4_ administration in MS-NASH mice on WDF aggravated liver injury and centrilobular fibrosis, pathology scores evaluated by the pathologist did not present enhanced pathology in steatosis, inflammation, ballooning and overall NAS scores from H&E images (Fig. 2g), nor the fibrosis score from PSR images (Fig. 2h, left). However, a quantitative AI measurement of fibrotic area by computer analysis software (Halo) from PSR images showed a significantly greater fibrosis area in the CCl_4_ (~ 8%) treated animals than those on CD or WDF (~ 2%) without CCl_4_ (Fig. 2h, right).

#### Low dose CCl_4_ (0.08 mL/kg), twice weekly for 8 weeks

**Fig. 3:**
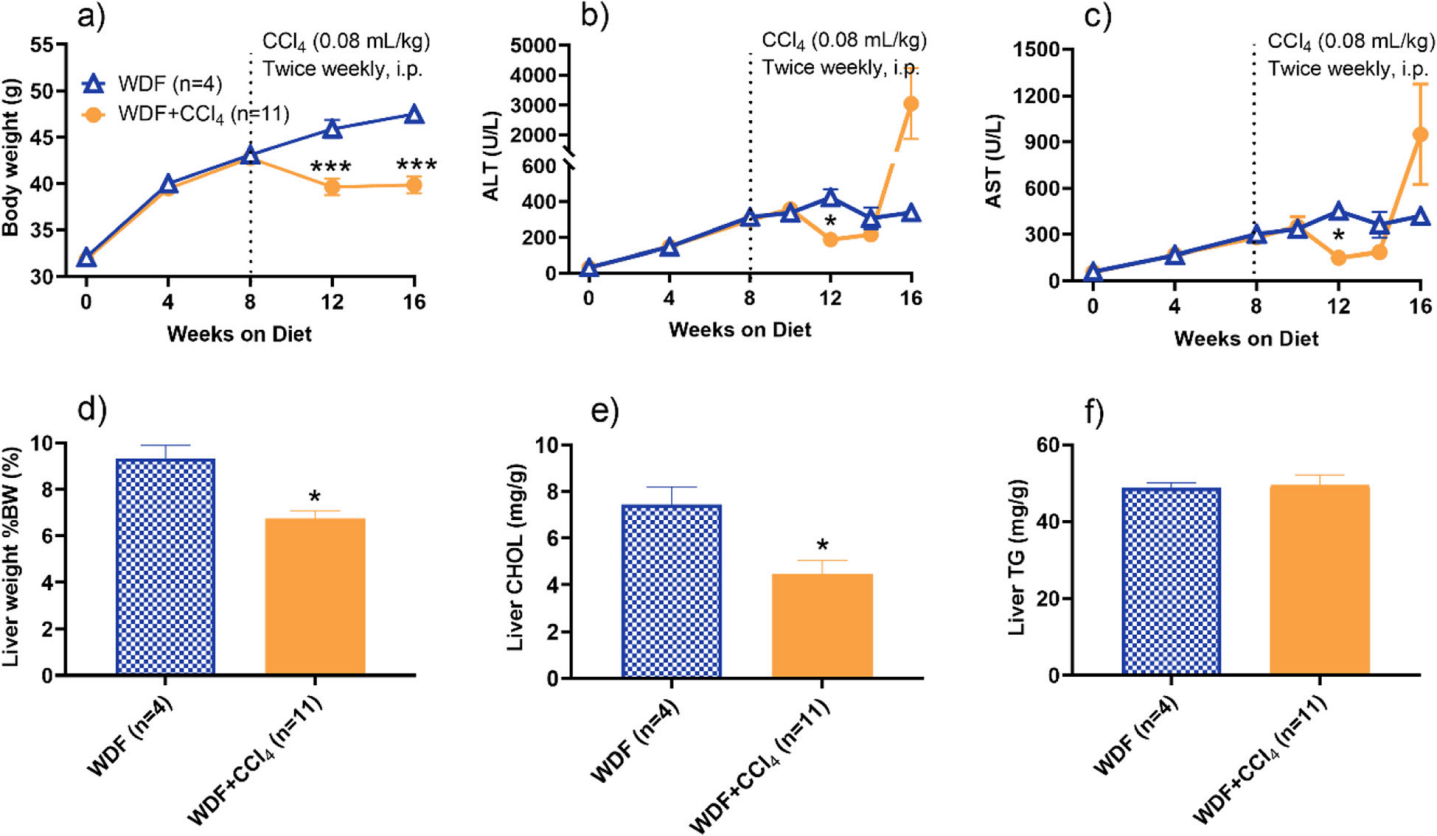
Effects of low dose CCL_4_ (0.08 mL/kg) twice weekly for 8 weeks in MS-NASH mice fed Western diet supplemented with fructose (WDF). Top panel: (**a**) Body weights; serum (**b**) ALT and (**c**) AST levels before and after low dose CCL_4_. In-life AST and AST levels at weeks 11, 12 and 14 were measured ~ 72 h; and the terminal one at week 16 measured ~ 24 h, after CCl_4_ administration. Bottom Panel: Liver (**d**) weight; (**e**) cholesterol; and (**f**) triglycerides measured at the end of the study. Data presented as mean ± SEM. * p < 0.05, *** p < 0.005, WDF vs. WDF + CCl_4_ group by Holm-Sidak t-test

To further reduce the toxicity of CCl_4_, a separate experiment was performed with the dose of CCl_4_ being lowered to 0.08 mL/kg BIW in MS-NASH mice on WDF. Compared to the WDF group without CCl_4_, low dose CCl_4_ prevented weight gain (Fig. 3a), and resulted in a transit reduction of serum ALT (Fig. 3b) and AST (Fig. 3c) only at week 12, but quick rebound to a much higher level than WDF group, although it was not as dramatically high compared to the mice receiving high dose CCl_4_. The liver weight (Fig. 3d) and contents of cholesterol (Fig. 3e), but not triglycerides (Fig. 3f), were significantly reduced by CCl_4_.

**Fig. 4:**
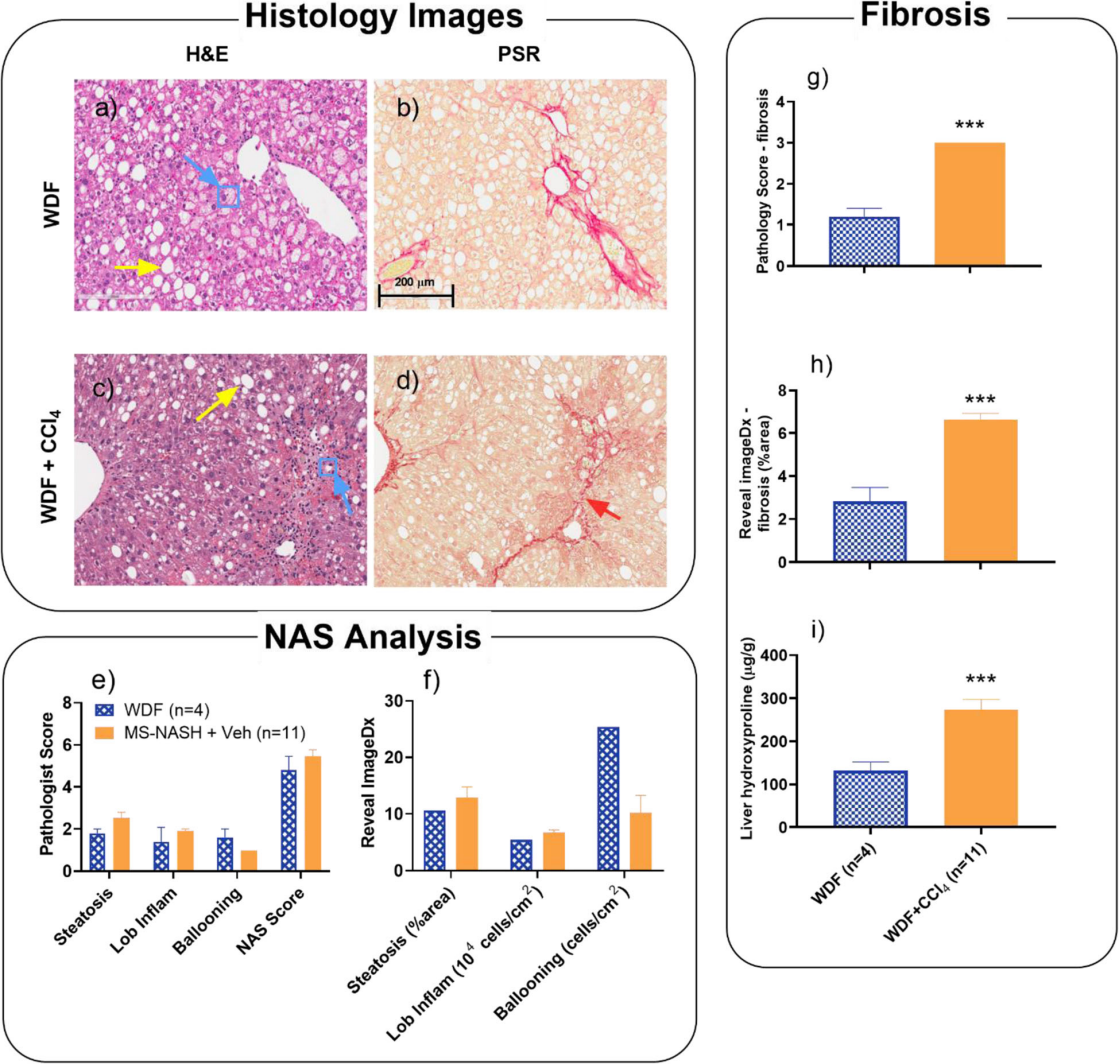
Histopathology in Western diet supplemented with fructose (WDF) fed MS-NASH mice treated with low dose CCL_4_ (0.08 mL/kg) for 8 weeks. Left panel: Representative images of H&E and PSR staining from MS-NASH mice on WDF. Yellow arrows indicate macrovesicular vacuolation steatosis; blue arrows indicate typical microvesicular ballooning within the blue square; and red arrows indicate fibrosis. (**a** and **b**) Without CCl_4_. (**c** and **d**) With CCl_4_. Bottom panel: Hepatosteatosis analysis from H&E images. (**e**) Semi quantification of pathology scores (0–3), lobular inflammation (0–3), ballooning (0–2), and NAFLD activity (0–8). (**f**) Quantitative histology analyzed as percentage of steatosis area, and cell counts of inflammation and hepatic ballooning by Reveal ImageDx software. Right panel: Fibrosis analysis from PSR images and live contents. (**g**) Fibrosis score by pathologist (0–4). (**h**) Fibrosis area by ImageDx. (**i**) Live hydroxyproline contents by biochemistry. Data presented as mean ± SEM. *** p < 0.005, WDF vs. WDF + CCL_4_ group using Holm-Sidak t-test

MS-NASH mice on WDF without CCl_4_ showed similar histopathological characteristics as reported earlier [22], with significant steatosis (Fig. 4a) and moderate fibrosis (Fig. 4b). However, MS-NASH mice on WDF treated with CCl_4_ presented persisting hepatosteatosis and hepatocyte ballooning degeneration in H&E stained images (Fig. 4c), as well as typical perisinusoidal and periportal fibrosis, along with enhanced bridging fibrosis in PSR stained images (Fig. 4d). Quantitative analysis revealed that low dose CCl_4_ significantly aggravated liver fibrosis (Figs. 4g, h, i) with little influence on liver steatosis (Figs. 4e & f) measured by both pathology score and computerized imaging analysis (Reveal ImageDx) and consistent with liver hydroxyproline contents.

### Therapeutic effects of Obeticholic acid (OCA) in MS-NASH or C57BI/6 mice on WDF treated low dose CCL_4_ (0.08 mL/kg, BIW) for 8-weeks

**Fig. 5:**
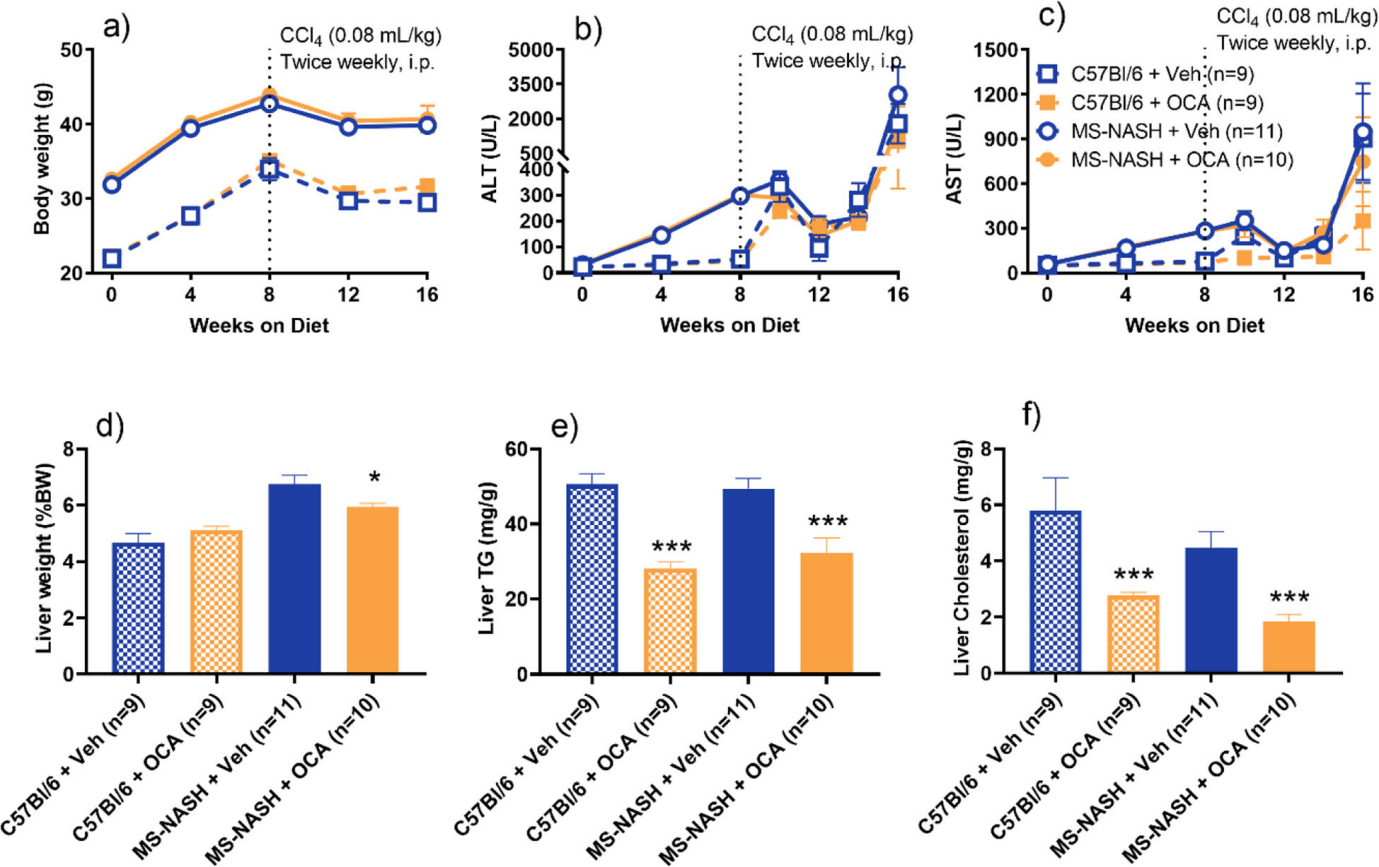
Therapeutic effects of Obeticholic acid (OCA, 30 mg/kg, QD) in Western diet supplemented with fructose (WDF) fed MS-NASH or C57BI/6 mice treated low dose CCl_4_ (0.08 mL/kg) twice weekly for 8 weeks. Top panel: (**a**) Body weight; serum (**b**) ALT and (**c**) AST. Bottom panel: (**d**) Terminal liver weight; and liver contents of (**e**) triglycerides and (**f**) cholesterol. Data represented as mean ± SEM. * p < 0.05, *** p < 0.005, Veh. vs OCA groups by Holm-Sidak t-test

Obeticholic Acid (OCA, 30 mg/kg, QD) or vehicle was administered orally in MS-NASH or C57Bl/6 mice fed WDF and treated with low dose CCl_4_ (0.08 mL/kg) twice weekly for 8 weeks. Compared to the vehicle groups, OCA had no significant effect on body weight (Fig. 5a) and serum ALT level (Fig. 5b) in both MS-NASH and C57Bl/6 mice, but lowered AST only in C57Bl/6 in mice (Fig. 5c). However, OCA significantly reduced liver contents of triglycerides (Fig. 5e) and cholesterol (Fig. 5f) in both MS-NASH and C57Bl/6 mice, and reduced liver weight only in MS-NASH mice (Fig. 5d).

**Fig. 6:**
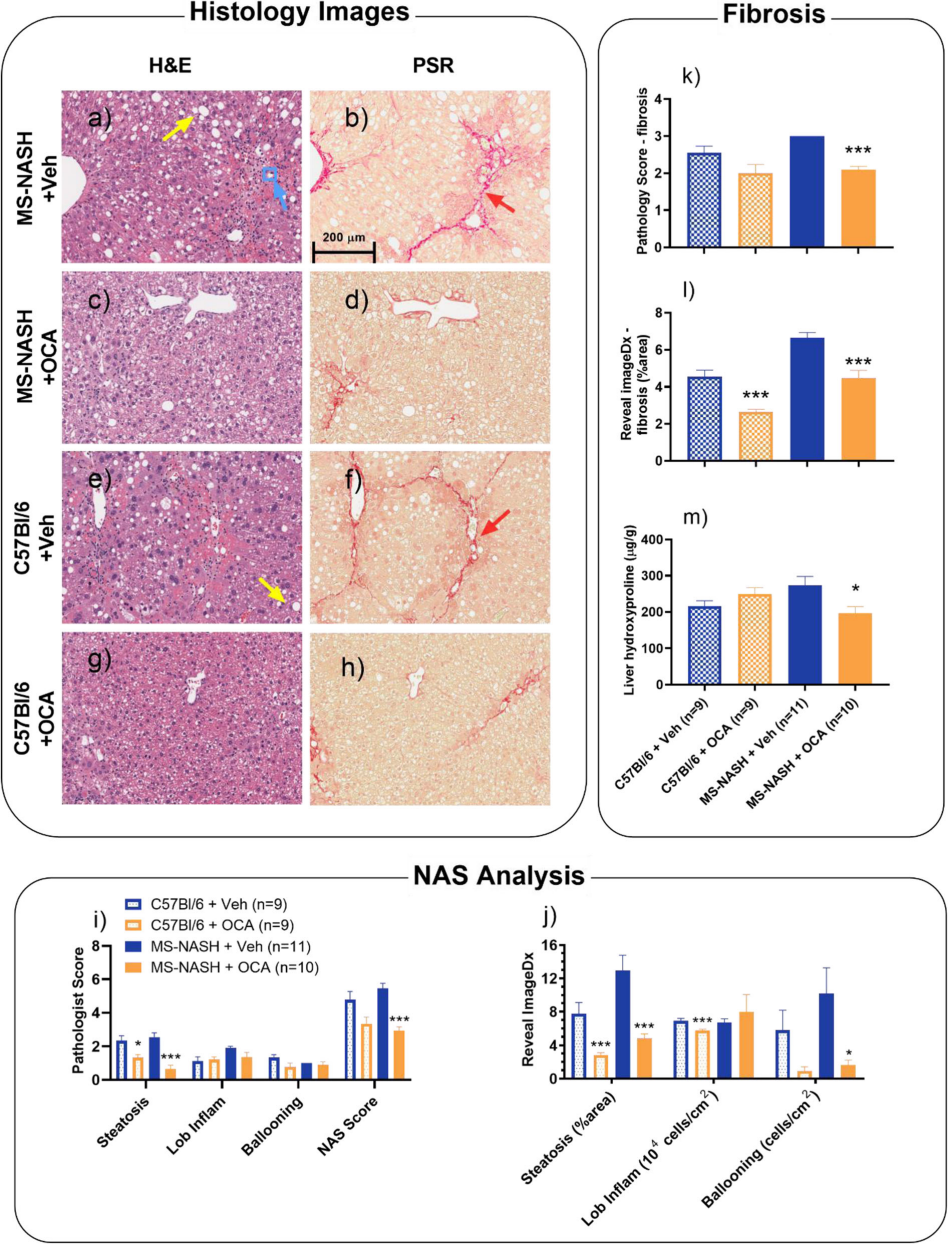
Histopathology of Obeticholic acid (OCA, 30 mg/kg, QD) treatment on Western diet supplemented with fructose (WDF) fed MS-NASH or C57BI/6 mice under low dose CCl_4_ (0.08 mL/kg) twice weekly for 8 weeks. Left panel: Representative images of H&E and PSR staining in mice on WDF treated with CCl_4_. Yellow arrows indicate steatosis, blue arrows indicate typical microvesicular ballooning within the blue square, and red arrows indicate fibrosis. MS-NASH mice with (**a** and **b**) vehicle or (**c** and **d**) OCA. C57Bl/6 mice with (**e** and **f**) vehicle or (**g** and **h**) OCA. Bottom panel: Hepatosteatosis analysis from H&E images. (**i**) Semi quantification of Pathology scores (0–3), lobular inflammation (0–3), ballooning (0–2), and NAFLD activity (0–8). (**j**) Quantitative histology analyzed as percentage of steatosis area, and cell counts of inflammation and hepatic ballooning by Reveal ImageDx software. Right panel: Fibrosis analysis from PSR images and liver contents. (**k**) Fibrosis score by pathologist (0–4). (**l**) Fibrosis area by ImageDx. (**m**) Live hydroxyproline contents by biochemistry. Data represented as mean ± SEM. * p < 0.05, *** p < 0.005, Veh VS OCA group using Holm-Sidak t-test

Representative histopathology images showed less lipid vacuoles and alleviated bridging fibrosis from OCA (Figs. 6c, d, g & h) compared to vehicle (Figs. 6a, b, e & f) groups. These observations were confirmed by both semi-quantification of pathologist scoring and computerized AI quantification (Reveal ImageDx) that treatment of OCA significantly reduced liver steatosis (Figs. 6i & j) and fibrosis (Figs. 6k & l) with more robust effects in MS-NASH compared to C57Bl/6 mice. The robust anti-fibrotic effect of OCA was further confirmed by significantly reduced liver hydroxyproline contents only in MS-NASH but not in C57Bl/6 mice (Fig. 6m).

### Survival rate in MS-NASH and C57BI/6 mice on WDF and treated high and low dose of CCL_4_

**Fig. 7:**
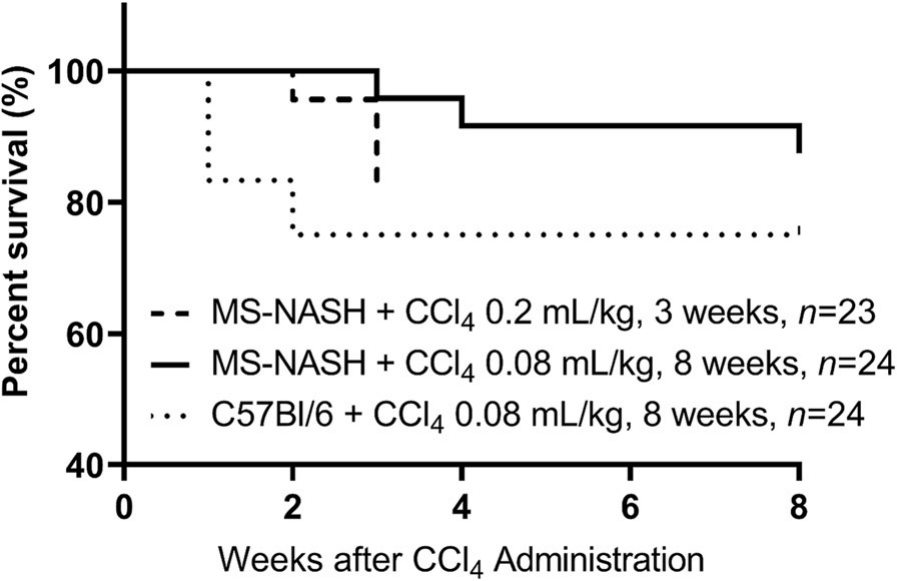
Comparison of survival rates in Western diet supplemented with fructose (WDF) fed MS-NASH or C57BI/6 mice under CCl_4_ twice weekly

The majority of mortality occurred in the first 3 weeks of CCl_4_ administration in both MS-NASH and C57Bl/6 mice. High dose CCl_4_ caused death in ~ 20% MS-NASH mice within the first 3 weeks, leading to early termination of the first experiment (Fig. 7). The survival rate in MS-NASH mice under lower dose CCl_4_ surpassed those under high dose CCl_4_ in the first 3 weeks and reached 87.5% at the end of entire 8-week experimental duration. The survival rate tended to be lower in 57Bl/6 than MS-NASH mice with low dose CCl_4_. However, this trend was not statistically significant among all the groups.

### Correlation of imagining analysis between the pathology score and computerized quantification

**Fig. 8:**
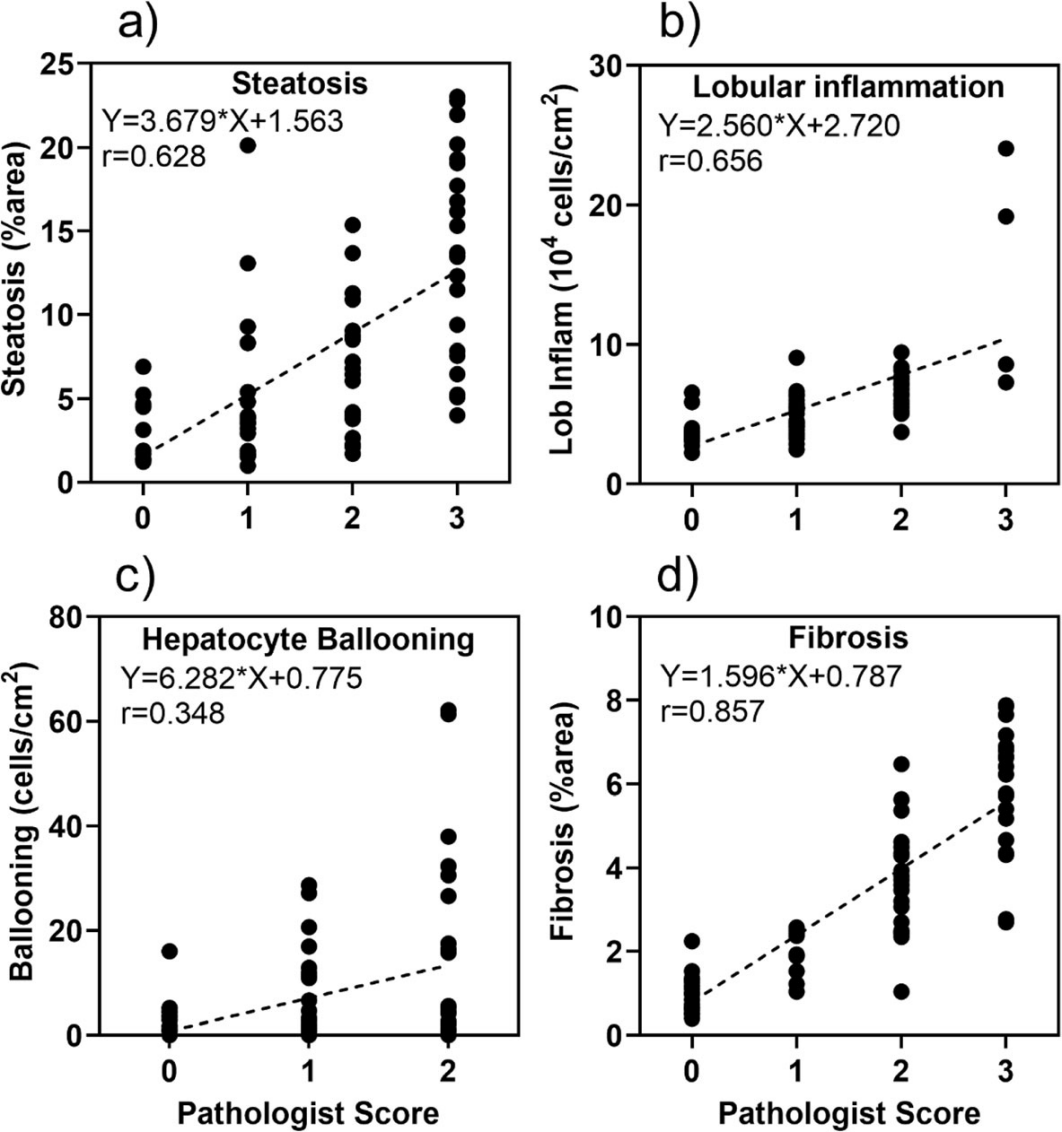
Correlation between Pathology scores and Reveal ImageDx analysis. Correlations between pathology scores for (**a**) steatosis; (**b**) lobular inflammation; (**c**) hepatocyte ballooning; and (**d**) fibrosis and Reveal ImageDx quantification by simple linear correlation with Pearson’s coefficients. All the Pearson correlation coefficient r values are statistically significant

A simple linear correlation analysis was performed on 4 aspects of histology readouts between pathologist scoring and quantitative image analysis with ImageDx software. Steatosis (Fig. 8a), lobular inflammation (Fig. 8b), hepatocyte ballooning degeneration (Fig. 8c) and fibrosis (Fig. 8d) scores all showed significant correlations between the 2 independent analyses.

## Discussion

The present data confirmed our previous report that MS-NASH mice possess all features of metabolic diseases [19, 20] and NAFLD/NASH when on WDF [22], however, with a relatively long induction duration (~ 20 weeks), and moderate liver fibrosis (pathology score ~ 1). Low dose CCl_4_ (0.08 mL/kg) accelerated the progression of NASH in ~ 8 weeks and exacerbated liver fibrosis by raising the pathology score to ~ 4, but did not significantly affect other liver pathology criteria of the NAS score in MS-NASH mice on WDF. Consistent with preclinical and clinical reports [17, 30–32], OCA treatment reduced NASH pathology in this model and presented more robust therapeutic benefits observed previously in MS-NASH mice on WDF only [22]. The liver hydroxyproline contents further confirmed histopathological observation of CCl4 enhanced liver fibrosis in MS-NASH mice on WDF, which can be significantly reduced by OCA treatment. Thus, MS-NASH mice fed WDF with low dose CCl_4_ administration provides a useful translational model for testing novel therapeutics targeting NASH/NAFLD.

The pathogenesis of NAFLD/NASH is initiated by systemic dysmetabolism, leading to lipid accumulation, hepatosteatosis, hepatocyte ballooning [4], inflammatory cell infiltration, increased oxidative stress [6, 7], hepatocyte injury, fibrosis, etc. [8–10] . CCl_4_ is a known liver toxin causing direct hepatocyte injury, leading to liver fibrosis, cirrhosis and carcinoma, which often requires relatively high doses from 0.2 to 5 mL/kg [23]. In order to avoid aggressive direct hepatocyte injury overshadowing development of hepatosteatosis, a critical component of NAFLD/NASH in MS-NASH mice, CCl_4_ at 0.2 mL/kg was selected in the first experiment. This dose caused excessive animal death in the first 3 weeks, likely due to acute liver toxicity evidenced by significant weight loss, reduction of food and caloric intake, and massive elevation of ALT and AST, without having sufficient time to develop significant change in liver pathology measured by steatosis, ballooning and overall NAS or fibrosis score. When the dose of CCl_4_ was reduced to 0.08 mL/kg, the mortality rate was significantly reduced with elevation of ALT and AST at the levels not as high as that observed in the high dose CCl_4_ group, although it still inflicted a low level general toxicity evidenced by reduction in bodyweight, liver weight and cholesterol content, which however is tolerable for most of the animals. On the other hand, the low dose CCl_4_ seems not affecting hepatic triglycines accumulation induced by WDF.

Although several noninvasive imaging methods have been used to assist diagnoses of NAFLD/NASH in clinical and preclinical research [33–36], histopathological examination is still the gold standard, especially to differentiate NASH from simple steatosis [29, 37, 38]. In preclinical research with rodent models, postmortem histopathological examination of liver tissue is still a commonly used method, in which the NAFLD Activity Score (NAS) is semi quantitatively evaluated by pathologists for assessment of NAFLD to distinguish steatosis from NASH [29]. NAS provides a composite score based on the degree of steatosis, lobular inflammation, and hepatocyte ballooning with a score < 2 signifying non-NASH; and ≥ 5 signifying clinical NASH. In the present study, liver histopathology in MS-NASH mice on WDF exhibited persisting macrovesicular steatosis, hepatocyte ballooning degeneration, and inflammatory cell infiltration with the NAS scores ~ 5, meeting the qualification for presenting a NSAH model (Figs. 2 and 4). The NAS does not include fibrosis score, with the latter being reported separately on a scale from 0 (without fibrosis) to 4 (cirrhosis) [29]; NAS and fibrosis scores do not always correlate with each other [28]. In the present experiment, the fibrosis scores were relatively low, ~ 1.2 in MS-NASH mice on WDF for 16 weeks, which was raised to ~ 3 by low dose CCl_4_ for 8 weeks (Figs. 4 and 6), but not by high dose CCl_4_ for 3 weeks (Fig. 2h). We suspect a longer duration may be needed for fibrosis development to the level detectable by the pathology scoring system due to its low sensitivity and resolution. The criteria of pathology score for fibrosis from 1 to 3 also depends on zonal distribution of fibrotic findings, with a score of 1 being periportal fibrosis; a score of 2 being periportal and perisinusoidal fibrosis; and a score of 3 being bridging fibrosis between multiple fibrotic areas. MS-NASH mice on WDF develop perilobular fibrosis initially, while CCl_4_-induced liver fibrosis exhibits centrilobular distribution characteristics. As showed in the present results, a correct combination of these 2 insults (WDF + CCl_4_) can generate aggravated fibrosis at multiple zones and accelerated bridging between areas.

The semiquantitative pathology scoring system with manually slide reading by pathologists is not only time-consuming and labor-intensive, but also subjective with person to person deviation, and lacks resolution and sensitivity to detect subtle difference in different animal models, different disease stages, and subtle changes by therapeutic intervention. With development of computerized imaging analysis and machine learning technology, commercial software is now available for automatic quantification of histopathology images, including liver pathology for NASH research [32, 39, 40]. As shown in Fig. 2h, a semi-quantitative fibrosis score of 1 may be too low to distinguish any differences among treatment groups. However, quantitative image analysis can measure the relative area of fibrosis over the entire section as ~ 2% in the control group, and increased to ~ 8% by high (Fig. 2h) or low (Fig. 4g and h, 6 k&l) dose CCl_4_ in MS-NASH mice on WDF. Furthermore, the present data also showed high correlation between a computer quantification and the pathology scores for steatosis, ballooning, inflammation and fibrosis (Fig. 8). Thus, the computerized image analysis is a valid method that is more efficient and consistent, providing higher sensitive and less subjective quantification of histopathology changes in NASH research.

The farnesoid X receptor agonist OCA has been used in preclinical treatment for diet [17] or chemical [41] induced liver fibrosis and NASH. The present data demonstrated a more robust efficacy of OCA treatment in MS-NASH mice on both WDF and CCl_4_ compared to those on WDF alone [22], which might be due to the model with a more robust liver fibrosis or OCA with dual alleviation to both diet/chemical-induction.

Mitochondrial metabolism dysregulation has been implicated in NALFD pathogenesis and progression with reduced capacity to compensate for increased oxidative stress, a key factor in hepatic injury and fibrosis, although the precise etiology of disease merits further investigation [42]. Accumulating evidence suggests that therapeutically targeting the mechanisms leading to mitochondrial dysfunction may have benefits for patients with liver disease [43–46]. The present survival analysis data showed that MS-NASH mice better tolerated CCl_4_ associated mortality compared to C57Bl/6 mice, which could be attributed to higher catalase activity to oxidative stress, thus, reducing oxidative DNA damage in MS-NASH mice reported by Boland et al. [47].

The present data also demonstrate that the degree of NASH pathology measured by both NAS and fibrosis scores from the pathologist and computer quantification appeared to be higher in MS-NASH than C57Bl/6 mice on WDF and CCl_4_, indicating that C57Bl/6 mice may require longer NASH induction time and have less hepatocyte ballooning degeneration, consistent with the view that MS-NASH mouse is a superior NASH model with more prominent hepatosteatosis pathology and metabolic disorders.

## Conclusions

CCl_4_ at 0.08 mL/kg reduced NASH induction time and exacerbated liver fibrosis formation while maintaining the other pathologic changes such as hepatic lipid accumulation, steatosis, ballooning, inflammation, etc. in MS-NASH mice on WDF. The NAFLD/NASH phenotypes in this model can also be ameliorated by the treatment of OCA. This yields a translational animal model of NASH that closely mimics human disease, therefore has been used in the pharmaceutical industry for testing novel therapeutic drugs in treatment of NASH and metabolic disorders.

## Data Availability

The datasets generated and analyzed during the current study are not publicly available due to potential commercial misuse but are available from the corresponding author on reasonable request.

## Abbreviations

AI: Artificial intelligence
ALT/AST: Aspartate/Alanine aminotransferase
BIW: Twice weekly
CD: Control diet
CCl4: Carbon; tetrachloride
DIO: Diet-induced obesity
H&E: Hematoxylin and Eosin
HCC: Hepatocellular carcinoma
i.p.: intraperitoneal
MS-NASH mouse: Metabolic syndrome-NASH
NAFLD/NASH: Nonalcoholic fatty liver disease/steatohepatitis
NAS: NAFLD activity score
OCA: Obeticholic acid
p.o.: oral
PSR: Picro Sirius Red
QD: Once daily
WDF: Western diet supplemented with fructose
